# Association of maternal mineral status with the risk of preterm birth: a retrospective cohort study

**DOI:** 10.3389/fnut.2024.1329720

**Published:** 2024-05-10

**Authors:** Sumiao Hong, Nan Jiang, Guankai Lin, Quqing Wang, Xiaoyang Xu, Xinrui Shi, You Zhou, Xiaoting Wen, Baochang Sun, Hexing Wang, Min Huang, Jiwei Wang, Na Wang, Yue Chen, Qingwu Jiang

**Affiliations:** ^1^Department of the Obstetrics, The People's Hospital of Pingyang, Wenzhou, China; ^2^Key Lab of Health Technology Assessment of Ministry of Health, School of Public Health, Fudan University, Shanghai, China; ^3^Wenzhou Center for Disease Control and Prevention, Wenzhou, China; ^4^Faculty of Medicine, School of Epidemiology and Public Health, University of Ottawa, Ottawa, ON, Canada

**Keywords:** mineral, preterm birth, micronutrients, gestational age, retrospective study

## Abstract

**Background:**

There has been a gradual increase in the proportion of preterm birth in China during the past several decades. Maternal malnutrition is a significant determinant for preterm birth. Nevertheless, comprehensive studies investigating serum mineral levels during pregnancy associated with preterm birth remain scarce. This study aims to assess the associations between maternal serum mineral levels and the risk of preterm birth.

**Methods:**

This retrospective cohort study of 18,048 pregnant women used data from a tertiary hospital in China from January 2016 to December 2022. Demographic data and serum mineral concentrations in the second and third trimesters of mothers were collected from the hospital information system. Analysis was performed using restricted cubic splines and logistic regression models.

**Results:**

The proportion of preterm birth in this study was 6.01%. Phosphorus [P for overall = 0.005; P for nonlinear = 0.490; OR (95%CI) = 1.11 (1.04, 1.18)] and chlorine [P for overall = 0.002; P for nonlinear = 0.058; OR (95%CI) = 1.11 (1.03, 1.19)] showed a significant positive correlation with preterm birth in a linear fashion. Furthermore, serum levels of potassium (P for nonlinear <0.001), sodium (P for nonlinear = 0.004), and magnesium (P for nonlinear <0.001) exhibited non-linear relationships with the risk of preterm birth.

**Conclusion:**

Serum levels of some minerals during pregnancy were associated with the risk of preterm birth among pregnant women. In addition to commonly recognized micronutrients such as folic acid, iron, and vitamin D, healthcare providers should also pay attention to the levels of these minerals during pregnancy.

## 1 Introduction

In the past decades, the general health status of Chinese women and children has been significantly improved (1), and the neonatal mortality rate has substantially reduced (2). However, the proportion of preterm birth in China increased from 5.36% in 1990 to 7.04% in 2015 (3). Preterm birth, defined as baby born before 37 weeks of gestation, is associated with an increased risk of a range of short-and long-term comorbidities in the neonate (4). Preterm birth not only imposes an economic and psychological burden on families, but also associated with enormous costs to health systems (5, 6).

Preterm birth is influenced by a variety of maternal and fetal characteristics, including maternal demographic characteristics, psychological factors, pregnancy status, nutritional status, and genetic factors (7). During pregnancy, a series of continuous physiological changes lead to increased maternal energy and micronutrients requirements to support optimal fetal growth and maternal health (8, 9). Hence, Maternal malnutrition can have profound effects on fetal development and pregnancy outcomes, including the occurrence of preterm birth (10, 11). Minerals, as important nutrition elements, play a variety of roles in the body, such as construction of bones, cofactor for the enzyme activity, and regulation of blood sugar (8, 12). Deficiency in minerals may lead to several consequences, including increased risk of pregnancy complications and perinatal mortality, fetal growth retardation, preterm birth, low birth weight, and infants being small for gestational age (13–15). Currently, numerous studies focuses on investigating common nutrient deficiencies such as folate, iron, and vitamin D (16–19), while some recent studies paid attention to minerals (20). For example, the disturbances of calcium(Ca)-phosphorus(P)-magnesium(Mg) homeostasis in pregnant women may lead to gestational hypertension by enhancing smooth muscle reactivity (21). In addition, enhanced uterine smooth muscle tone and uterine contractility may lead to the occurrence of preterm birth (21, 22). Sodium(Na) retention during pregnancy can lead to alterations in maternal blood pressure and weight, thereby influencing fetal growth (23). Reduced potassium(K) levels may induce premature contraction of the uterus, leading to preterm birth (24). Mg supplementation in early pregnancy could play a role in preventing preterm birth (25, 26). However, another study found that Mg supplementation during pregnancy did not improve maternal and neonatal health outcomes (27). The impact of mineral intake and its level in maternal blood on the risk of preterm birth remains inconclusive. Specifically, whether deficiencies or excess of some minerals have different effects on preterm birth needs to be further explored.

However, most previous studies have only assessed the minerals individually in relation to preterm birth. To the best of our knowledge, there is only one cohort study that comprehensively investigated the relationships between various maternal mineral levels and the risk of preterm birth, but the sample size of the study was small, including only 780 mother-offspring pairs (28). Besides, maternal minerals are transferred to the fetus through the placenta, with this transfer process experiencing rapid growth starting from the second trimester and reaches its maximum in the third trimester (29, 30). Therefore, the effects of maternal mineral levels on fetal growth and delivery may be most significant in the second or third trimester. Hence, we conducted a large retrospective epidemiological study to investigate the relationships between maternal serum mineral levels in the second and third trimesters of pregnancy and the occurrence of preterm birth.

## 2 Materials and methods

### 2.1 Study population

This study was a retrospective cohort study of 18,048 pregnant women at the People’s Hospital of Pingyang, Wenzhou City, Zhejiang Province, China between January 2016 and December 2022. The hospital serves as the primary provider of medical and healthcare services in Pingyang county, functioning as the sole tertiary hospital. A majority of expectant mothers in this region opt for this hospital for delivery. The hospital has an efficient information system that archives the demographic and clinical data of every patient. All participants in this study underwent pregnancy registration and examinations and had a singleton delivery. Pregnant women with missing information on mineral status or last menstrual period, age younger than 18 years, or intrauterine fetal death were excluded from this study. The study was approved by the Medical Research Ethics Committee of the School of Public Health, Fudan University (The international registry no. IRB00002408 and FWA00002399). The retrospective study used existing data and all the information on the personal identification was removed for the study. Informed consent was not sought, which was also approved by the Medical Research Ethics Committee of the School of Public Health, Fudan University.

### 2.2 Data collection and measurements

The demographic and clinical data were collected from the hospital information system. The primary outcome of this study was preterm birth, defined as birth before 37 weeks gestation. Gestational age was calculated by using the last menstrual period combined with ultrasound measurements. Mineral information was obtained from records of the latest antenatal examination prior to inpatient delivery, all in the second and third trimesters. Antecubital venous blood samples were collected from pregnant women who had fasted for more than 8 h using 5-mL BD vacutainer blood collection tube (Becton Dickinson, Franklin Lakes, NJ, United States). Fasting was required as the blood sample would also be utilized for a range of tests, including liver and renal function tests. After the blood samples were allowed to clot, it was centrifuged at 3500 rpm for 10 min. Following centrifugation, the serum samples were freed from the interference of hemolysis, jaundice and lipid turbidity. The levels of Na, K, and chlorine (Cl) were determined using the ion selective electrode method, Ca levels were determined using the arsenazo method, P levels were determined using the phosphomolybdate method, and Mg levels were determined using the enzymatic method. The corresponding data of minerals were obtained from the Architect c16000 clinical chemistry analyzer (Abbott Diagnostics, Abbott Park, United States).

### 2.3 Covariates

The covariates included age, parity (previous live births), amniotic fluid volume, hyperglycemia during pregnancy, hypertensive disorders of pregnancy, *in vitro* fertilization, and neonatal sex. Information on age and parity was recorded by the physician at the first pregnancy registration visit. Amniotic fluid volume was assessed using ultrasound measurements, including maximal vertical pocket and amniotic fluid index. Oligohydramnios was defined as maximal vertical pocket <2 cm or amniotic fluid index <5 cm. Polyhydramnios was defined as maximal vertical pocket >8 cm or amniotic fluid index >24 cm. Hyperglycemia during pregnancy including diabetes mellitus and gestational diabetes mellitus. Hypertensive disorders of pregnancy including chronic (preexisting) hypertension, gestational hypertension, preeclampsia, and eclampsia.

### 2.4 Statistical analysis

Statistical analysis was performed in R 4.2.3.^1^ Continuous variables were presented as mean (standard deviation, SD) or median (interquartile range, IQR), where appropriate. Categorical variables were presented as numbers and percentages. We examined the dose–response relationship between maternal mineral levels and preterm birth risk using restricted cubic splines with 4 knots. The number of knots used in the restricted cubic spline analysis was selected corresponding to the minimum Akaike information criterion. Given the non-direct interpretability of regression parameters obtained from regression splines (31), we employed binary logistic regression models to derive meaningful parameters for linear relationship outcomes. For non-linear relationship outcomes, we constructed 2-piecewise logistic regression models to calculate inflection points and analyze threshold effects. In the logistic regression model, we calculated the odds ratios (ORs) and 95% confidence intervals (CIs) for preterm birth in association with one standard deviation change of the mineral levels. Restricted cubic splines and logistic regression analyses were adjusted for age, parity (previous live births), amniotic fluid volume, hyperglycemia during pregnancy, hypertensive disorders of pregnancy, *in vitro* fertilization, and neonatal sex. A *p* value <0.05 was considered statistically significant.

## 3 Results

### 3.1 Characteristics of study participants

**Figure 1:**
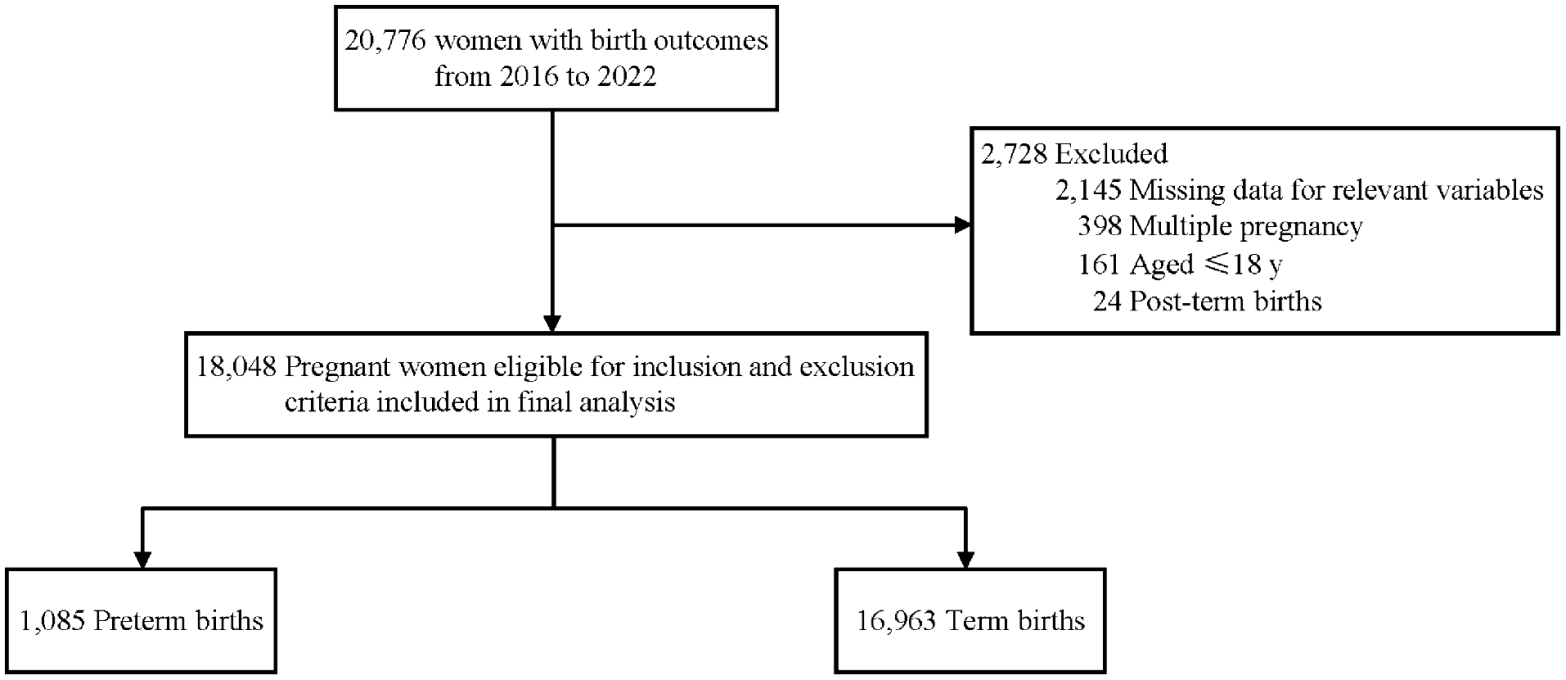
Flowchart of study participants.

A total of 18,048 mother-singleton infant pairs were included in this retrospective cohort study (Figure 1), with the mean age at delivery of 30.05 ± 4.80 years, of which 16.52% were advanced maternal age (Table 1). Among the participants, 38.25% of pregnant women were primiparous, 13.99% were diagnosed with hyperglycemia during pregnancy, and 3.83% were diagnosed with hypertensive disorder of pregnancy. The average serum level for all the pregnant women in the second or third trimesters was 2.20 ± 0.16 mmol/L for Ca, 3.87 ± 0.28 mmol/L for K, 1.24 ± 0.21 mmol/L for P, 137.41 ± 1.85 mmol/L for Na, 103.74 ± 2.53 mmol/L for Cl, and 0.79 ± 0.12 mmol/L for Mg. The median gestational age of childbirth was 39.0 weeks (38.0–40.0 weeks), and 6.01% of mothers experienced preterm birth.

**Table 1:** Characteristics of participants.

Characteristics	All participants
Participants, *n*	18,048
Maternal age, mean (SD), y	30.05 (4.80)
Maternal age group, *n* (%), y	
<35	15,066 (83.48%)
≥35	2,982 (16.52%)
Parity group, *n* (%)	
None	6,904 (38.25%)
1–2	11,049 (61.22%)
3 or more	95 (0.53%)
Amniotic fluid volume, *n* (%)	
Oligohydramnios	1,919 (10.63%)
Normal	15,710 (87.05%)
Polyhydramnios	419 (2.32%)
Hyperglycemia during pregnancy, *n* (%)	2,525 (13.99%)
Hypertensive disorders of pregnancy, *n* (%)	691 (3.83%)
*In vitro* fertilization, *n* (%)	331 (1.83%)
Neonatal sex	
Male	9,805 (54.33%)
Female	8,243 (45.67%)
Calcium, mean (SD), mmol/L	2.20 (0.16)
Potassium, mean (SD), mmol/L	3.87 (0.28)
Phosphorus, mean (SD), mmol/L	1.24 (0.21)
Sodium, mean (SD), mmol/L	137.41 (1.85)
Chlorine, mean (SD), mmol/L	103.74 (2.53)
Magnesium, mean (SD), mmol/L	0.79 (0.12)
Gestational age at delivery, median (IQR), wk	39.0 (38.0–40.0)
Preterm birth, *n* (%)	1,085 (6.01%)

SD, standard deviation; IQR, interquartile range.

### 3.2 Association between mineral levels and preterm birth

**Figure 2:**
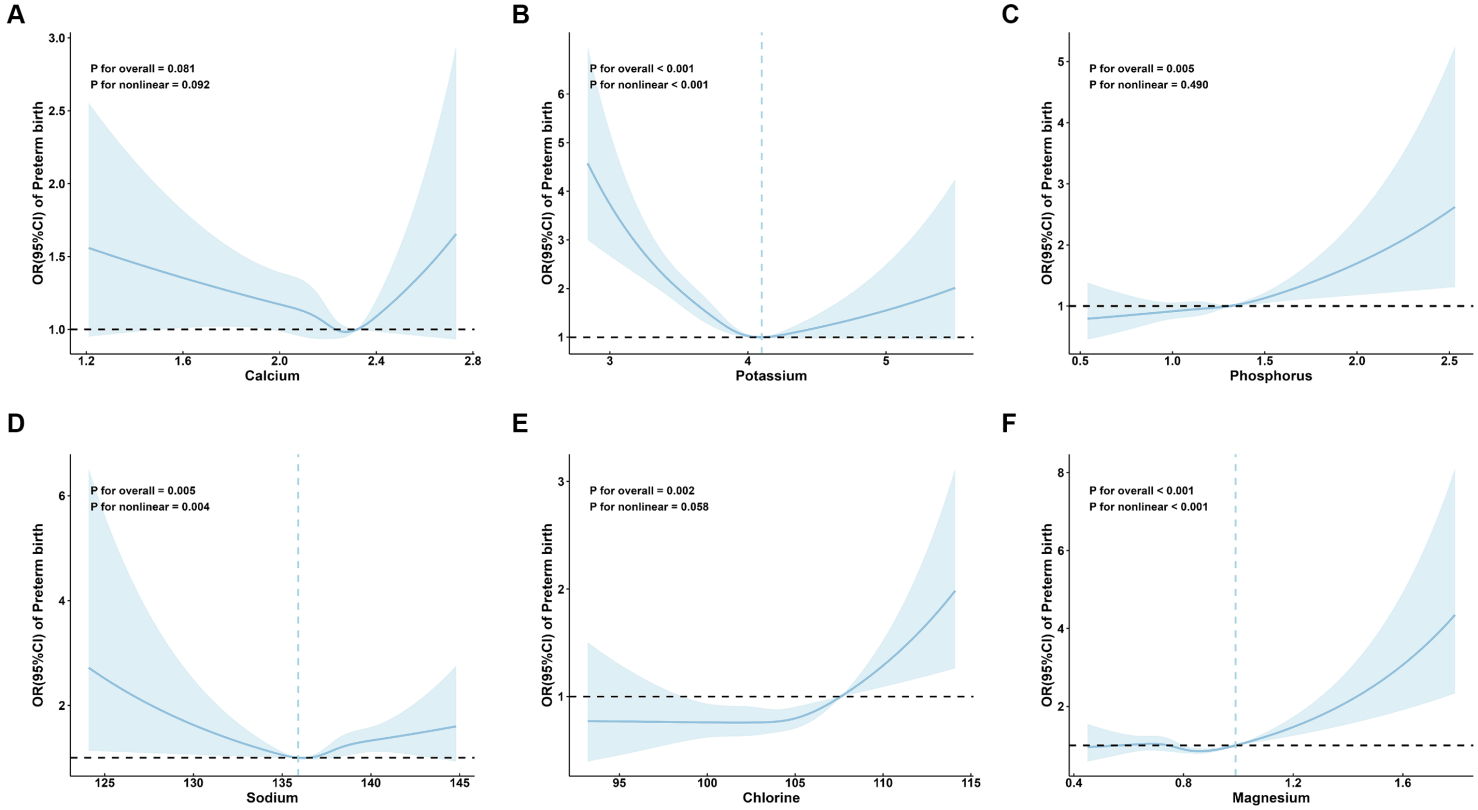
Adjusted* odds ratios and 95 % confidence intervals for preterm birth in association with maternal serum mineral levels in restricted cubic spline regression. **(A)** calcium; **(B)** potassium; **(C)** phosphorus; **(D)** sodium; **(E)** chlorine; and **(F)** magnesium. * Adjusted for age (continuous), parity, amniotic fluid volume, hyperglycemia during pregnancy, hypertensive disorders of pregnancy, in vitro fertilization, and neonatal sex.

No significant associations were observed between Ca and preterm birth (P for overall = 0.081; Figure 2A).

A significant linear positive correlation between P and preterm birth was demonstrated (P for overall = 0.005; P for nonlinear = 0.490; OR (95%CI) = 1.11 (1.04, 1.18); Table 2; Figure 2C). Also, there is a significant linear positive correlation between Cl and preterm birth [P for overall = 0.002; P for nonlinear = 0.058; OR (95%CI) = 1.11 (1.03, 1.19); Table 2; Figure 2E].

**Table 2:** Adjusted* odds ratios (ORs) and 95% confidence intervals (95% CIs) for preterm birth in association with maternal serum mineral levels in multivariable logistic regression.

Serum mineral level	Inflection point (mmol/L)	Group (mmol/L)	OR (95%CI)	*p* value
Potassium (per 1 SD)^a^	4.10	≤ 4.10	0.70 (0.65, 0.76)	<0.001
		>4.10	1.20 (1.01, 1.42)	0.042
Phosphorus (per 1 SD)	NA	NA	1.11 (1.04, 1.18)	0.001
Sodium (per 1 SD)^a^	135.90	≤135.90	0.80 (0.68, 0.95)	0.009
		>135.90	1.15 (1.04, 1.26)	0.004
Chlorine (per 1 SD)	NA	NA	1.11 (1.03, 1.19)	0.004
Magnesium (per 1 SD)^a^	0.99	≤0.99	0.93 (0.85, 1.02)	0.116
		>0.99	1.39 (1.19, 1.62)	<0.001

a Piecewise logistic regression.

*Adjusted for age (continuous), parity, amniotic fluid volume, hyperglycemia during pregnancy, hypertensive disorders of pregnancy, in vitro fertilization, and neonatal sex.

Restricted cubic spline regression analysis revealed that serum levels of K (P for overall <0.001; P for nonlinear <0.001; Figure 2B), Na (P for overall = 0.005; P for nonlinear = 0.004; Figure 2D), and Mg (P for overall <0.001; P for nonlinear <0.001; Figure 2F) exhibited nonlinear relationships with the risk of preterm birth. Specifically, the restricted cubic spline demonstrated a U-shaped association of both K and Na with preterm birth, as well as an L-shaped nonlinear relationship between Mg and preterm birth.

The inflection point of K was 4.10 mmol/L. On the left side of the inflection point, the risk of preterm birth decreased with increasing maternal serum K level (OR: 0.70; 95% CI: 0.65, 0.76) (Table 2). On the right side, the risk of preterm birth increased with increasing maternal serum K levels (OR: 1.20; 95% CI, 1.01, 1.42) (Table 2). The inflection point of Na was 135.90 mmol/L. Na was a protective factor for preterm birth on the left side of the inflection point (OR: 0.80; 95% CI: 0.68, 0.95) (Table 2), and a risk factor on the right side (OR:1.15; 95% CI: 1.04, 1.26) (Table 2). The inflection point of Mg was 0.99 mmol/L. On the right side of the inflection point, the risk of preterm birth increased with increasing maternal serum Mg levels (OR: 1.39; 95% CI: 1.19, 1.62), but not significant on the left side (OR: 0.93; 95% CI: 0.85, 1.02) (Table 2).

### 3.3 Subgroup analysis of preterm birth by maternal age, parity, and neonatal sex group

**Figure 3:**
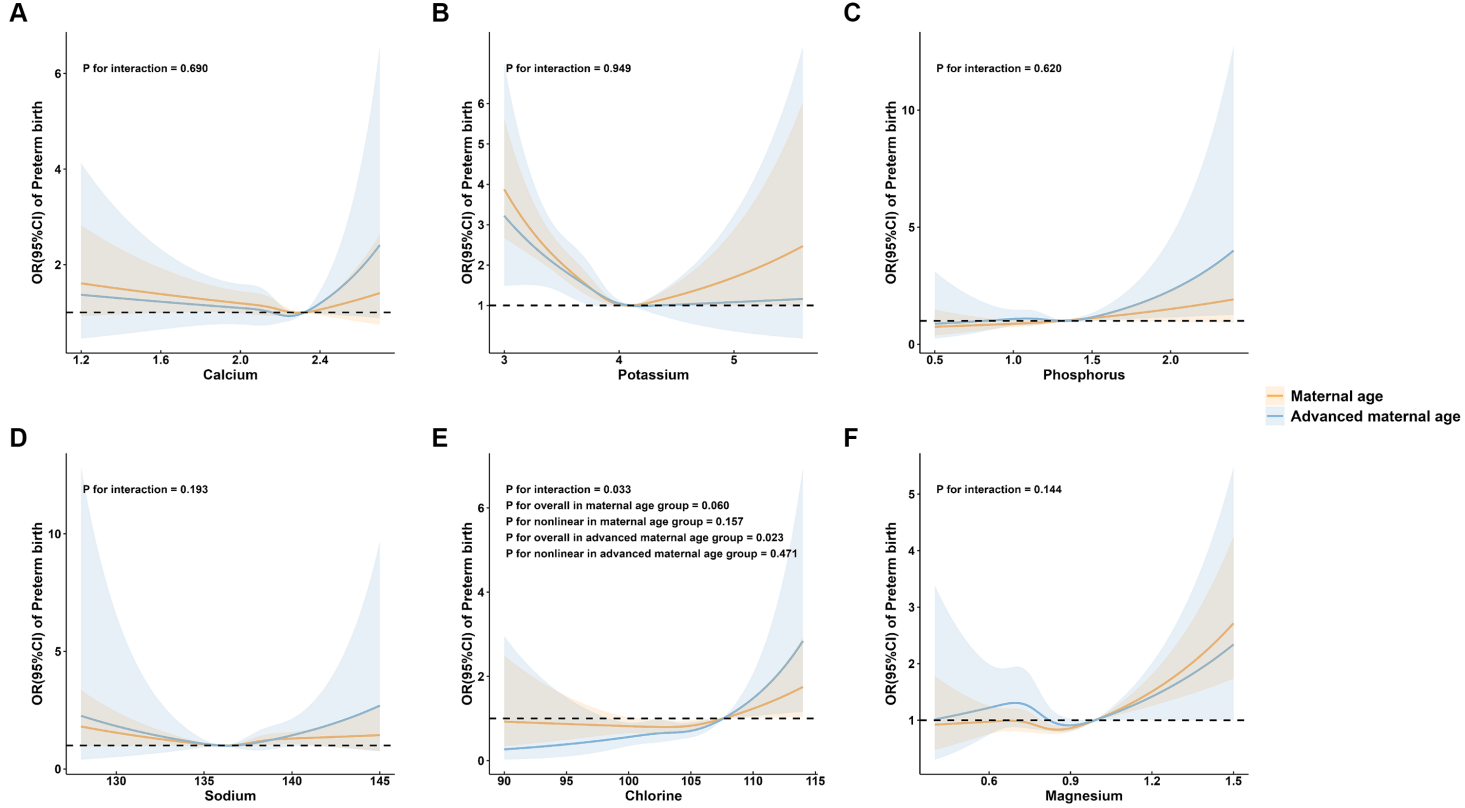
Subgroup analysis of preterm birth by maternal age group. **(A)** calcium; **(B)** potassium; **(C)** phosphorus; **(D)** sodium; **(E)** chlorine; and **(F)** magnesium.

**Figure 4:**
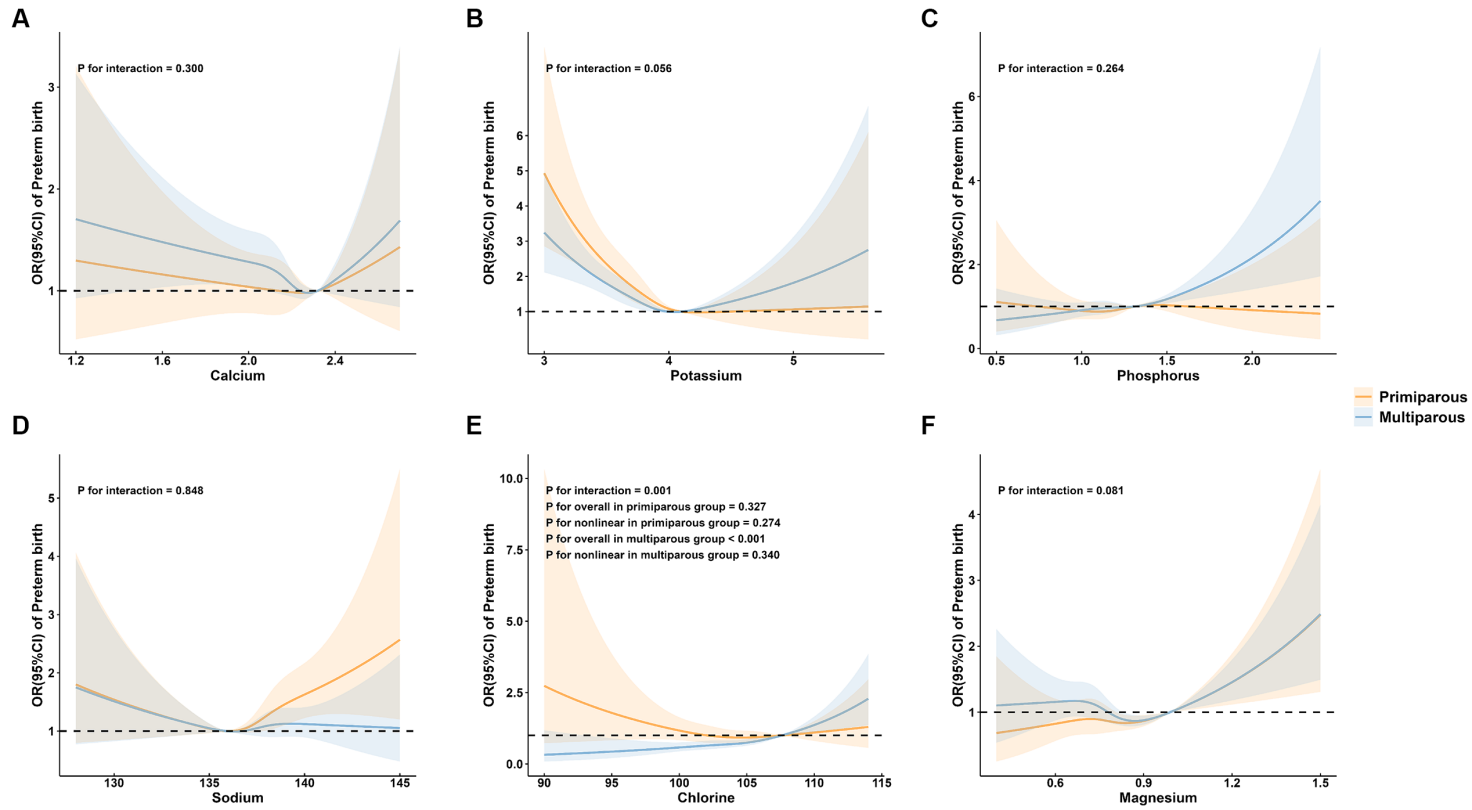
Subgroup analysis of preterm birth by maternal parity group. **(A)** calcium; **(B)** potassium; **(C)** phosphorus; **(D)** sodium; **(E)** chlorine; and **(F)** magnesium.

**Figure 5:**
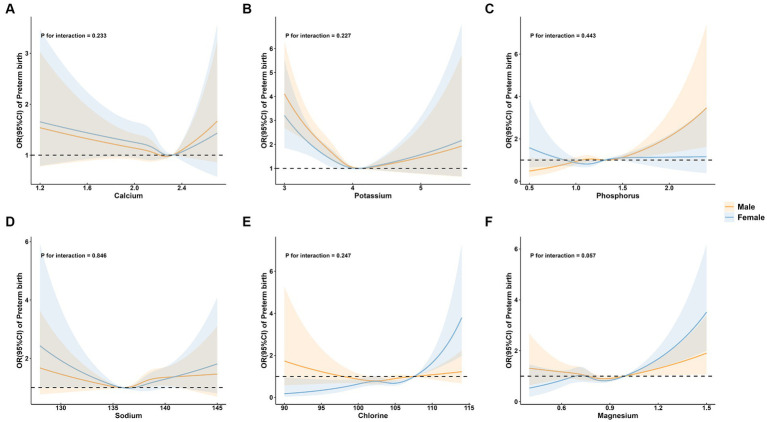
Subgroup analysis of preterm birth by neonatal sex group. **(A)** calcium; **(B)** potassium; **(C)** phosphorus; **(D)** sodium; **(E)** chlorine; and **(F)** magnesium.

To further explore the association between maternal mineral levels and preterm birth, stratified analyses by maternal age, parity, and neonatal sex were performed (Figures 3–5). The restricted cubic spline plots illustrated that maternal serum Cl levels interacted with maternal age (P for interaction = 0.033; Figure 3E) and parity (P for interaction = 0.001; Figure 4E). Among women under age of 35, no significant association was observed between Cl and preterm birth (P for overall = 0.060; P for nonlinear = 0.157). Conversely, a linear positive correlation was observed between Cl and preterm birth among women of advanced maternal age (P for overall = 0.023; P for nonlinear = 0.471). Similarly, there was a linear positive correlation between Cl and preterm birth among multiparous women (P for overall <0.001; P for nonlinear = 0.340), whereas no association was observed among primiparous women (P for overall = 0.327; P for nonlinear = 0.274).

## 4 Discussion

We conducted a large-scale retrospective study to investigate the relationships between the serum concentrations of several minerals in pregnant women and the risk of preterm birth. We observed that maternal serum levels of K, P, Na, Cl, and Mg were shown distinct associations with preterm birth after adjusting for various confounding variables.

Among the minerals in this study, both low and high levels of K were found to be independent risk factors for preterm birth. K is an essential mineral for the body that maintains fluid balance, helps maintain blood pressure, and regulates muscle contraction, among other functions. K channels in the smooth muscle layer of the uterus play an important role in regulating myometrial tone and controlling uterine contractions during pregnancy. Studies have demonstrated that K^+^ channel dysfunction or decreased expression would diminish the repolarization current of mesenchymal stem cells, thereby inducing premature uterine contractions and preterm birth (24). In addition, elevated K levels during pregnancy have been found to be associated with increased risks of gestational diabetes mellitus, preeclampsia, and cardiovascular disease, which may impair normal fetal growth and result in preterm birth (32–34). Currently, there is no defined threshold for normal levels of K during pregnancy (32). The findings of this study suggest that maintaining K levels at a moderate level of about 4.10 mmol/L is appropriate, which falls within the normal range of 3.5–5.5 mmol/L for adults (35). Nonetheless, further research is required to determine the optimal range of K levels for pregnant women.

In this study, it was found that elevated maternal serum P and Cl levels would increase the risk of preterm birth. P is crucial for numerous bodily functions such as blood clotting, nerve function, muscle movement, and bone strengthening (36). However, excess of P during pregnancy can be harmful to both mother and fetus. In excessive amounts, it inhibits the absorption of other essential minerals in the body, severely disrupts the hormonal regulation of phosphate, Ca, and vitamin D, leading to tissue hardening in the body, anemia, impaired kidney function, and bone loss (36, 37). Cl plays a vital role in electrolyte balance and the maintenance of kidney and muscle functions (38). Elevated serum Cl levels can lead to a range of health issues, including dehydration and metabolic acidosis (38). The subgroup analysis revealed interactions of Cl with maternal age and parity. The liner positive correlations between Cl and preterm birth only appeared in women of advanced maternal age and multiparous women. This suggests that women of advanced maternal age and multiparous women are more sensitive to the harm associated with elevated serum Cl levels. Therefore, it is crucial to pay more attention to preventing excessive serum Cl levels in these groups during pregnancy. However, the specific mechanisms by which P and Cl contribute to preterm birth in different populations require further exploration.

Our findings showed a U-shaped relationship between maternal serum Na levels and the risk of preterm birth. As the most important electrolyte in extracellular fluid, Na regulates the osmolality in the extracellular space and plays a crucial role during pregnancy (39). Decreased maternal Na levels during pregnancy may lead to an enhancement of the renin angiotensin system, which promotes cardiovascular and renal injury mechanisms (40, 41). Consequently, this could hinder the development of fetal cardiovascular or metabolic systems, contributing to impaired fetal growth and an increased risk of low birth weight and preterm birth (39, 40). Furthermore, studies have shown that elevated Na levels in the blood of pregnant women are related to an increased risk of developing high blood pressure and preeclampsia during pregnancy, which can lead to intrauterine growth retardation and preterm birth (42–44). Besides, researchers hypothesize that excessive Na intake during pregnancy may affect fetal nutrition supply by influencing placental blood flow, thereby impacting fetal growth and leading to preterm birth (42). Our study is consistent with previous studies showing the importance of keeping maternal Na levels within an appropriate range during pregnancy. During pregnancy, on one hand, serum Na levels in pregnant women may mildly decrease due to increased circulatory volume (45). On the other hand, various conditions such as hemorrhage, overheating, hyperemesis, and diarrhea can lead to sodium deficiency and altered appetite for salt (46). Therefore, food, especially salt, as the main source of serum Na for pregnant women, should be strictly controlled during pregnancy to prevent harm from deficiency or excess.

Mg is a mineral that is beneficial for regulating almost every system in the body (27). Previous studies on Mg supplementation during pregnancy have yielded inconsistent results. Some studies suggested that supplementing Mg during pregnancy may reduce the incidence of various pregnancy complications, including fetal growth restriction and preeclampsia (47). However, a review incorporating ten studies indicates no evidence supporting the improvement of maternal and neonatal health outcomes through Mg supplementation during pregnancy (27). In this study, we found a plateau in the relationship between Mg and preterm birth at low levels, but as Mg levels continued to increase above a certain threshold, the risk of preterm birth also increased. Consistent with our results, the toxic effects of excess Mg on cardiovascular complications, apnea, and coma during pregnancy have been demonstrated in multiple studies (48, 49). Interestingly, our results suggest that below a certain threshold, changes in Mg concentration may not significantly impact the occurrence of preterm birth. Further high-quality evidence is still needed to confirm the optimal range of Mg levels during pregnancy and explore its impact on maternal and infant health in the future.

There are several limitations of our study. First, some potential risk factors were not incorporated due to the lack of corresponding data in the hospital information system, such as smoking status, maternal BMI, and usage of nutritional supplementation. Second, each mineral has different distributions in different organs and systems. This study only measured the concentrations of minerals in serum. Third, this study only included maternal serum mineral data collected in the second and third trimesters since only a small proportion of individuals had their mineral levels measured during early pregnancy. Moreover, we did not explore the associations of mineral levels in different trimesters with preterm birth, given that only 668 pregnant women had mineral data in the second trimester of pregnancy, which may result in insufficient statistical power due to the small sample size. The associations between mineral levels in different trimesters of pregnancy and the risk of preterm birth should be investigated in future prospective studies.

## 5 Conclusion

Our study demonstrated significant associations between some maternal mineral levels and the occurrence of preterm birth. There was a significant positive association of P and Cl with preterm birth, and a non-linear relationship between K, Na and Mg and preterm birth. Healthcare providers should pay a closer attention to the levels of these mineral elements during pregnancy, beyond the commonly recognized micronutrients such as folate, iron, and vitamin D. Monitoring mineral levels during antenatal care can help early identification of potential deficiencies or imbalances that may contribute to adverse pregnancy outcomes. In addition, further large-scale, high-quality prospective studies are needed to explore the underlying mechanisms and validate the best range of minerals for achieving the optimal maternal and fetal health.

## Data availability statement

The raw data supporting the conclusions of this article will be made available by the authors, without undue reservation.

## Ethics statement

The studies involving humans were approved by Medical Research Ethics Committee of the School of Public Health, Fudan University (the international registry no. IRB00002408 and FWA00002399). The studies were conducted in accordance with the local legislation and institutional requirements. The ethics committee/institutional review board waived the requirement of written informed consent for participation from the participants or the participants’ legal guardians/next of kin because the retrospective study used existing data and all the information on the personal identification was removed for the study. Informed consent was not sought, which was also approved by the Medical Research Ethics Committee of the School of Public Health, Fudan University.

## Author contributions

SH: Data curation, Formal analysis, Writing – original draft. NJ: Data curation, Formal analysis, Writing – original draft. GL: Resources, Supervision, Writing – review & editing. QW: Investigation, Methodology, Writing – review & editing. XX: Resources, Visualization, Writing – review & editing. XS: Investigation, Software, Writing – review & editing. YZ: Methodology, Resources, Writing – review & editing. XW: Data curation, Software, Writing – review & editing. BS: Resources, Writing – review & editing. HW: Software, Validation, Writing – review & editing. MH: Supervision, Writing – review & editing. NW: Project administration, Resources, Writing – review & editing. JW: Methodology, Project administration, Supervision, Writing – review & editing. YC: Methodology, Writing – review & editing. QJ: Resources, Supervision, Writing – review & editing.

## Footnotes

^1^ http://www.R-project.org
